# High-Risk Features and Utility of Computed Tomography-Fluoroscopy-Guided Biopsy as a Diagnostic Tool

**DOI:** 10.1016/j.jaccas.2024.102925

**Published:** 2024-12-18

**Authors:** Abdullah H. Ghunaim, Nitish K. Dhingra, Sonja Kandel, Patrik Rogalla, Robert James Cusimano

**Affiliations:** a Division of Cardiac Surgery, Toronto General Hospital, University of Toronto, Toronto, Ontario, Canada; b Joint Department of Medical Imaging, Toronto General Hospital, University of Toronto, Toronto, Ontario, Canada

**Keywords:** cardiac tumor, CT-guided biopsy, myxoma, sarcoma

## Abstract

Left atrial masses are most commonly diagnosed as myxomas. When clinicians doubt the diagnosis, a biopsy is warranted. However, this can be very difficult with left-sided tumors. We present a 71-year-old woman with a presumed left atrial myxoma with features concerning for a malignant lesion. She underwent a computed tomography–fluoroscopy-guided percutaneous biopsy, revealing a diagnosis of malignant spindle cell neoplasm, thus avoiding surgery and allowing for early treatment.

## Graphical Abstract

**Figure:**
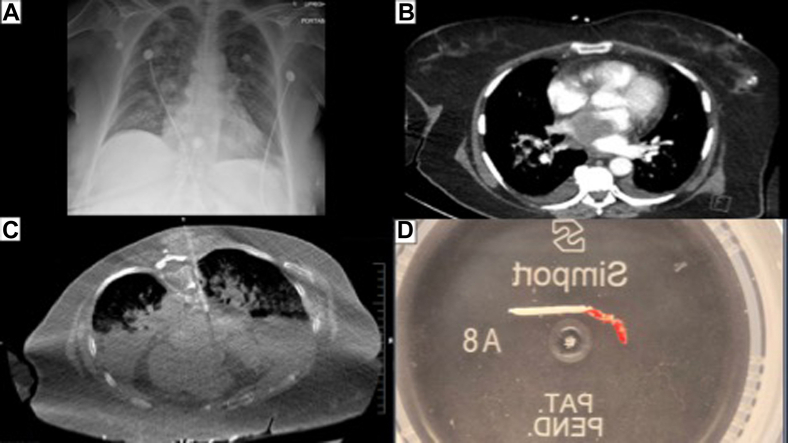

A 71-year-old woman presented to the emergency department with a 2-day history of falls, cough, nausea, diarrhea, vomiting, and malaise. An electrocardiogram revealed complete heart block. A chest x-ray (Figure 1A) showed airspace opacification consistent with pulmonary edema. A subsequent chest computed tomography (CT) (Figure 1B) uncovered a mass within the left atrium (4.2 × 4.1 cm) and bilateral airspace disease, which was worse on the right. Transesophageal echocardiogram showed a large irregular-shaped mass, heterogeneous in echotexture (4.6 × 5.1 cm), and occupying at least 50% to 60% of the left atrial volume. The mass was attached to the interatrial septum but extended inferiorly to the base of the left atrium near the atrioventricular node, reaching superiorly to the left atrial appendage. Signs of obstruction were noted in both upper pulmonary veins. A diagnosis of myxoma was made, and the patient was referred for a surgical intervention.

**Figure 1:**
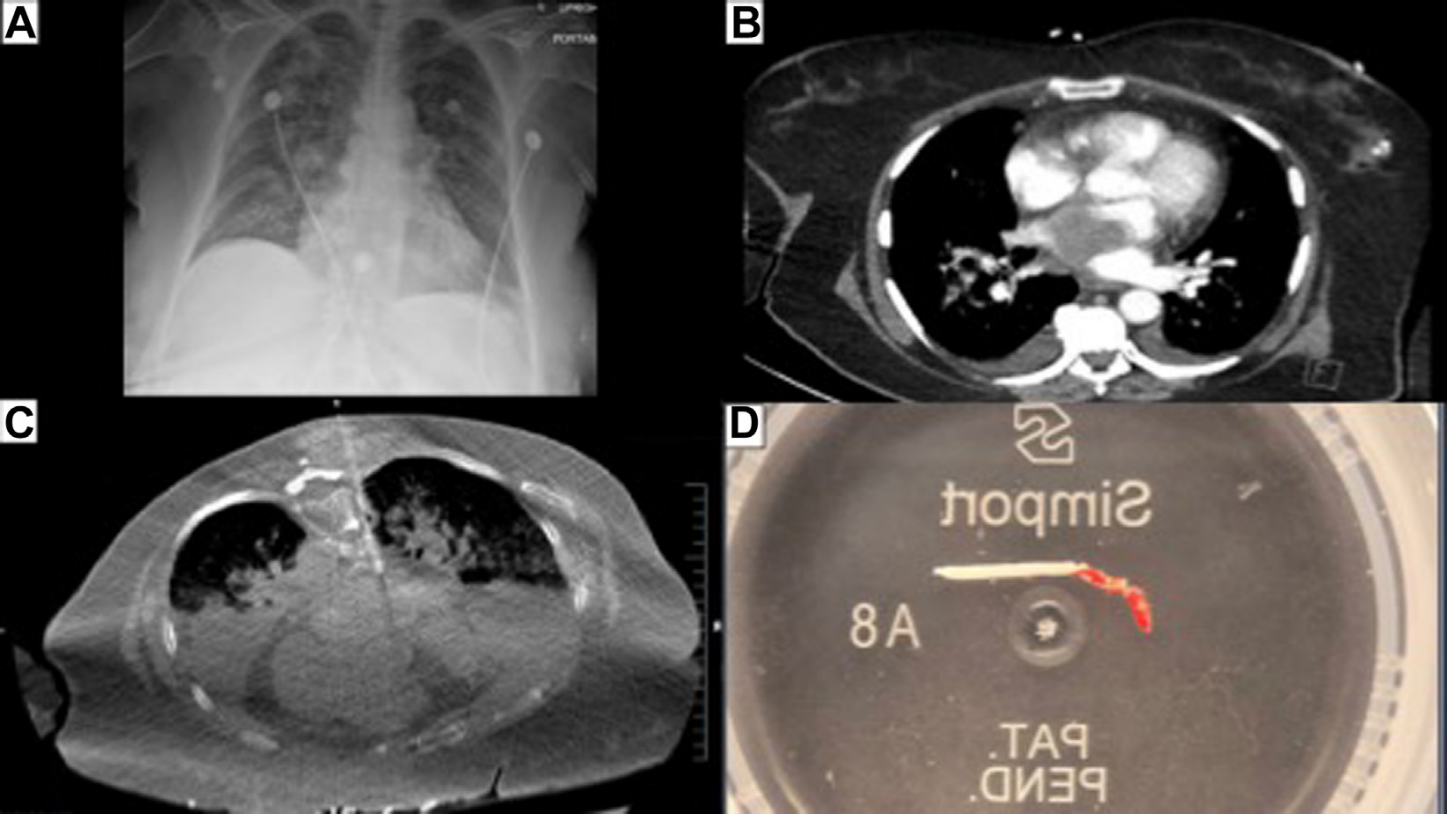
Chest X-Ray, Computed Tomography, and Mass Specimen (A) The initial chest x-ray demonstrating bilateral pulmonary edema. (B) Subsequent contrast-enhanced computed tomography (CT) reveals the tumor in the left atrium. (C) CT–fluoroscopy-guided percutaneous biopsy. (D) Specimen of the mass.

However, because of the pulmonary edema, bilateral superior pulmonary vein involvement, and the nonclassic location of the tumor, the concern for malignancy was raised. Given that an operation would necessitate bilateral pulmonary and cardiac resection, we considered a CT–fluoroscopy-guided percutaneous biopsy in consultation with our interventional radiology team. To protect the esophagus and lung, the right paravertebral space was dissected by injecting 40 mL of diluted lidocaine-contrast material solution through a blunt 17-gauge coaxial needle. An 18-gauge biopsy gun was then negotiated under CT–fluoroscopy guidance into the tumor while avoiding penetration of the perfused left atrial lumen as mapped on the contrast-enhanced planning CT (Figure 1C). A 2-cm core was obtained (Figure 1D) and fixed in formalin.

Histopathology demonstrated a morphologically undifferentiated malignant spindle cell neoplasm with MDM2 and CDK4 expression, favoring a diagnosis of cardiac undifferentiated pleomorphic sarcoma (ie, intimal sarcoma). Because the lesion was considered too extensive for primary resection and the patient was deemed too ill for chemotherapy, radiotherapy was chosen as the most appropriate treatment option. A permanent pacemaker was implanted for the complete heart block, allowing for safer treatment. Informed consent was obtained from the patient for publication.

Although many types of masses can be found in the left atrium, cardiac myxoma is the most common primary neoplasm of the heart and is found in the left atrium 80% of the time.^1^^,^^2^ Secondary (ie, metastatic) cardiac tumors are 20-fold more common than primary cardiac tumors.^1^ When confronted with a presumed benign myxoma in the left atrium, suspicion of an alternative diagnosis should be raised when abnormal, so-called high-risk features of the tumor are present. An unusual location of the tumor may serve as the first hint. Immobility and penetration of the heart and obstruction or involvement of the pulmonary vasculature causing pulmonary edema are further clues. Diagnostics are usually limited to surgery and transvenous biopsies, which can be dangerous in high-risk patients or low yield in unfavorable anatomy.

Our center has pioneered CT–fluoroscopy-guided percutaneous cardiac biopsy when other approaches are limited or unsuccessful in obtaining tissue.^3^ This technique has heralded a new era in the diagnosis of high-risk cardiac tumors and has changed the approach for such complex lesions.

## Funding Support and Author Disclosures

The authors have reported that they have no relationships relevant to the contents of this paper to disclose.

## References

[1] Bussani R., Castrichini M., Restivo L. (2020). Cardiac tumors: diagnosis, prognosis, and treatment. Curr Cardiol Rep.

[2] Basso C., Valente M., Poletti A., Casarotto D., Thiene G. (1997). Surgical pathology of primary cardiac and pericardial tumors. Eur J Cardiothorac Surg.

[3] Rogalla P., O'Brien C., Pakkal M. (2024). CT-fluoroscopy guided percutaneous biopsy of cardio-pericardial masses. Can Assoc Radiol J.

